# CD70 in Thymic Squamous Cell Carcinoma: Potential Diagnostic Markers and Immunotherapeutic Targets

**DOI:** 10.3389/fonc.2021.808396

**Published:** 2022-01-25

**Authors:** Jumpei Kashima, Tsunekazu Hishima, Yusuke Okuma, Hirotoshi Horio, Masumi Ogawa, Yukiko Hayashi, Shin-ichiro Horiguchi, Toru Motoi, Tetsuo Ushiku, Masashi Fukayama

**Affiliations:** ^1^ Department of Pathology, Tokyo Metropolitan Cancer and Infectious diseases Center Komagome Hospital, Tokyo, Japan; ^2^ Department of Pathology, Graduate School of Medicine, The University of Tokyo, Tokyo, Japan; ^3^ Department of Thoracic Oncology and Respiratory Medicine, National Cancer Center, Tokyo, Japan; ^4^ Department of Thoracic Surgery, Tokyo Metropolitan Cancer and Infectious diseases Center Komagome Hospital, Tokyo, Japan

**Keywords:** thymic carcinoma, CD70, CD27, immunohistochemistry, tumor-infiltrating lymphocyte

## Abstract

CD70 – a ligand protein of CD27 on lymphocytes – is expressed in a large spectrum of malignancies. It is an attractive target for antibody-based therapy and several clinical trials are currently being conducted. However, there is no evidence regarding the expression of CD70 and its relationship with expression of programmed death ligand-1 (PD-L1) and CD27+ tumor-infiltrating lymphocytes (TIL) in formalin-fixed paraffin-embedded (FFPE) tissues of thymic tumors. FFPE tissues of thymic squamous cell carcinoma (TSCC) (operative specimens, n = 31; biopsy specimens, n = 11), thymoma (n = 60), thymic carcinoid (n = 3), and lung squamous cell carcinoma (LSCC) (n = 30) were analyzed immunohistochemically. Immunoreactivity for CD70 was semi-quantitatively scored according to the proportion of positive tumor cells. Moreover, the densities of CD27-positive intratumoral TIL (iTIL) and stromal TIL of TSCC were assessed and survival was compared. Most TSCC cases (87%; 27/31) were CD70-positive. In contrast, all thymoma and thymic carcinoid cases were CD70-negative. In LSCC cases, CD70-positivity was significantly lower than TSCC cases (20%; 6/30). Biopsy and resected specimens obtained from the same patients demonstrated a consistent staining pattern (6/6 patients). The proportion of CD70-positive TSCC was comparable with those of CD5 (87%) and CD117 (90%). Correlation between CD70 and PD-L1 expression score was observed. There was no significant difference in survival between the CD70-high and CD70-low expression groups. Meanwhile, patients with CD27-positive iTIL-high tumors exhibited better survival than those with iTIL-low tumors. This tendency was weaker in the CD70-high subset. CD70 immunohistochemistry is useful in diagnosing TSCC. CD70 may prevent anti-tumor immunity *via* CD27. Immunotherapy targeting the CD70–CD27 axis may be a promising option for the treatment of TSCC.

## Introduction

CD70 belongs to the tumor necrosis factor family of proteins and acts as a ligand of CD27. Thymic epithelium and activated T-cells and B-cells are known to express CD70 (1), which promotes differentiation to effector or memory T-cells by expressing CD27 on lymphocytes (2). CD70–CD27 pathway is demonstrated to promote survival of FOXP3-positive regulatory T-cells (3). Hematological malignancies and solid tumors have also been shown to express CD70, leading to the development of antibodies against CD70 as therapeutic agents (4–6).

Thymic carcinoma is a rare disease, accounting for 14% of thymic epithelial tumors according to the International Thymic Malignancy Interest Group database (7). Thymic squamous cell carcinoma (TSCC), which accounts for the majority of thymic carcinoma cases, has some histological mimickers, such as type B3 thymoma and lung squamous cell carcinoma (LSCC). Immunohistochemical (IHC) analysis of CD5, CD117 (KIT), GLUT-1, and MUC1 is useful in differentiating TSCC from type B3 thymoma (8–19). In addition, CD5 and CD117 are helpful in distinguishing TSCC from LSCC (13, 14, 18).

Using IHC analysis in a limited number of frozen TSCC sections, we previously demonstrated that positive staining for CD70 was observed frequently in neoplastic epithelial cells of TSCC, unlike in those of thymoma and LSCC (20). Recently, an anti-CD70 antibody for formalin-fixed, paraffin-embedded (FFPE) tissues became commercially available; however, the diagnostic value of this antibody in TSCC is currently not well documented.

Programmed death ligand-1 (PD-L1) interacts programmed death-1 (PD-1) and inhibits antitumor immunity by T-cells. PD-L1 and PD-1 have become established targets of immunotherapy for various types of cancer. Several clinical trials of immune checkpoint inhibitors targeting PD-1/PD-L1 axis for thymic epithelial tumors have been conducted (21–23). These reagents seemed effective especially in thymic tumor with high-PD-L1 expression (21, 22).

Tumor-infiltrating lymphocytes (TIL) have been associated with favorable prognosis in various types of solid tumors (24–30). CD27-positive TIL are indicated to correlate with activated T-cell response in non-small cell lung cancer and renal cell carcinoma, both of which are known as CD70-expressing tumors (6, 31–33). However, currently, no studies examine the CD27+ TIL status in thymic carcinoma.

In the present study, we performed an IHC analysis for CD70 in TSCC and compared the results with those obtained in thymoma and LSCC. We also assessed the correlation between CD70 and PD-L1 expression in TSCC tissue. In addition, the correlation of the CD70-positivity of tumor cells and CD27-positive TIL on survival was analyzed.

## Materials and Methods

### Tissue Preparation and Patient Characteristics

TSCC (n = 31), thymoma (type A: n = 5, type AB: n = 20, type B1: n = 9, type B2: n = 9, type B3: n = 17), thymic carcinoid (n = 3), and LSCC (n = 30) resected at a single institution between 1987 and 2017 were analyzed retrospectively. Metastatic squamous cell carcinoma is excluded with clinical information. Biopsy specimens of TSCC (n = 11) and LSCC (n=12) were also analyzed. The diagnosis was based on hematoxylin and eosin staining of FFPE tissues. In addition, the diagnosis was reviewed and confirmed by two pathologists (J.K. and T.H.) according to the 4^th^ Edition of the World Health Organization Classification of Tumours of the Lung, Pleura, Thymus & Heart. The clinical characteristics of the patients were retrospectively obtained from the electronic medical records. Written informed consent was acquired from the patients. This study was approved by the Institutional Review Board of the Tokyo Metropolitan Cancer and Infectious Diseases Center Komagome Hospital (approval number: 2120).

### IHC Evaluation

FFPE tissues were subjected to IHC analysis. Sections (3.5 μm in thickness) were cut and stained with the avidin–biotin–peroxidase complex method using the ABC Elite Kit (Vector, Burlingame, CA, USA). The antibodies used in this study were CD70 (clone 301731, R&D Systems, Minneapolis, MN, USA; 1:50 dilution), CD5 (4C7, Leica Biosystems, Wetzlar, Germany; 1:70 dilution), CD117 (YR145, Epitomics, Burlingame, CA, USA; 1:2,000 dilution), PD-L1 (E1L3N, Cell Signaling, Danvers, MA, USA; 1:100 dilution), CD8(C8/144B, Nichirei, Tokyo, Japan; 1:100 dilution), FOXP3 (236A/E7, Abcam, Cambridge, UK; 1:150 dilution), and CD27 (HPA038936, Sigma-Aldrich, St. Louis, MO, USA; 1:50 dilution). To evaluate the positivity of each marker, the proportion of positive cells in random spots was estimated and graded as follows: CD70: score 0 (<1% positivity), score 1 (1–25% positivity), score 2 (26–50% positivity), score 3 (51–75% positivity), or score 4 (76–100% positivity); PD-L1: score 0 (<1% positivity), score 1 (1–49% positivity), or score 2 (50–100% positivity). Intratumoral TIL (iTIL) and stromal TIL (sTIL) were defined as infiltrating lymphocytes within tumor nests and tumor stroma, respectively. The average counts (cells per mm^2^) of CD8-, FOXP3-, and CD27-positive iTIL and stromal sTIL in triplicate high-magnification images obtained with Nikon DS-Ri2 (Nikon, Tokyo, Japan) and counted with NIS-Elements D Imaging Software (Nikon, Tokyo, Japan).

### Gene Expression Analysis

Analysis of gene expression was performed with real-time quantitative reverse transcriptase polymerase chain reaction (RT-PCR) using first-strand cDNA derived from RNA isolated from frozen tissues of TSCC (n = 12) and thymoma with few non-neoplastic lymphocytes (type A: n = 4, type B3: n = 5), as described previously (30). SYBR green sequence detection reagents (Applied Biosystems, Foster City, CA, USA) and sense and anti-sense primers were used. All reactions were performed in duplicate. The CD70 primers used were as follows: F, gctgctttggtcccattggtcg (exon 1); R, gaggtcctgtgtgattcagctg (exon 2/3 junction; 141-bp product). Reaction products were assayed on a LightCycler 480 Real-Time PCR Instrument (Roche Diagnostics, Rotkreuz, Switzerland) and the PCR product was measured in real time as the increase in SYBR green fluorescence. Data were analyzed using the LightCycler 480 Software version 1.5 (Roche Diagnostics). The CD70 copy number was standardized against the β-actin copy number in each sample.

### Survival Analysis

Survival was defined as the period from tissue acquisition of TSCC to death by any cause. We adopted only overall survival to see the prognostic value of CD70 and other immune-related factors in limited number of patients for two reasons. First, more than 40% of the patients enrolled in this study had stage IVa or IVb diseases, and many of them received palliative-intent surgery. Moreover, our study also enrolled patients with thymic carcinoma resected almost 30 years ago, when the following-up frequency after surgery and available images were heterogenous, which might have biased progression free survival. One case was excluded from survival analysis due to the lack of data. We compared the survival of patients with TSCC between two groups defined according to the CD70 IHC score (CD70-low: IHC score 0 or 1; CD70-high: score 2, 3, or 4), the number of CD27-positive iTIL (iTIL-low: iTIL<median; iTIL-high: iTIL≥median), and the number of CD27-positive sTIL (sTIL-low: sTIL<median; sTIL-high: sTIL≥median).

### Statistical Analysis

Tumor positivity, revealed by IHC (i.e., the proportion of tumors with a CD70 IHC score ≥1), was compared between TSCC and LSCC using Fisher’s exact test. Relation between CD70 and PD-L1 positivity score, and between CD8, FOXP3 and CD27-positive TIL were assessed using Spearman’s rank correlation test. Quantitative comparison of CD70 expression determined by RT-PCR between thymoma and TSCC, and comparison of TIL levels between CD70-high and low TSCC were performed using the Mann–Whitney U-test. Survival curves were generated using the Kaplan–Meier method. The univariate Cox proportional hazards regression model was employed to assess the hazards ratio according to age, while the log-rank test was used for categorical values (Masaoka–Koga staging, CD70-low/high, CD27-positive iTIL-low/high, and sTIL-low/high). Factors with p < 0.10 were included in the multivariate analysis. Subsequently, a multivariate Cox proportional hazards regression model was employed to estimate the relationship between variables and survival. A p-value < 0.05 denoted statistical significance. All calculations were performed using the R version 3.3.3 (Vienna, Austria).

## Results

### Patient Characteristics

The characteristics of patients with TSCC and thymoma are summarized in **Table 1**. A total of 31 patients with TSCC underwent surgical tumor resection. Of those, six patients also underwent biopsies prior to surgery. Notably, five patients with advanced disease underwent only biopsy. The cases with notable lymphocyte infiltration underwent Epstein-Barr virus encoded small RNA *in situ* hybridization to confirm the diagnosis of TSCC. None of the patients received immune checkpoint blockade after surgery.

**Table 1:** Patient characteristics.

	TSCC	Thymoma
**Male/female**	17/14	21/39
**Age, years (median, range)**	65.5, 41–83	58, 34–84
**Masaoka–Koga staging**	n (%)	n (%)
I	1 (3)	15 (25)
II	7 (23)	33 (55)
III	10 (32)	4 (7)
IVa	3 (10)	3 (5)
IVb	10 (32)	4 (7)
**Histological subtype**		(%)
A		5 (8)
AB		20 (33)
B1		9 (15)
B2		9 (15)
B3		17 (28)
**Neoadjuvant chemotherapy and/or radiotherapy**		
Yes	6 (19)	2 (3)
No	25 (81)	58 (97)

TSCC, thymic squamous cell carcinoma.

### IHC Analysis of CD70, CD5 and CD117

#### CD70 in the Healthy Thymus

**Figure 1:**
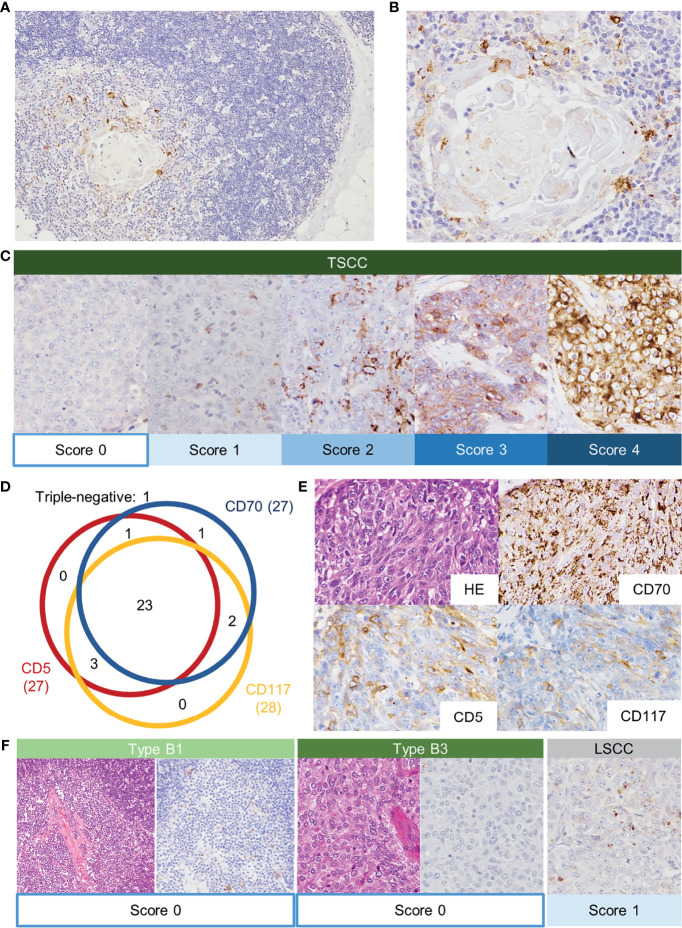
Normal adult thymus tissue stained with anti-CD70 antibody. Thymic medullary epithelial cells or dendritic cells were positive for CD70 **(A, B)**. Immunostaining of CD70 in thymic squamous cell carcinoma (TSCC) **(C)**. CD70-positivity was scored according to the proportion of positive tumor cells (score 0: <1%; score 1: 1–24%; score 2: 25–49%; score 3: 50–74%; score 4: 75–100%). Schematic summary of immunohistochemical analysis (D).The numbers indicate the positive cases of each staining. The numbers in the parentheses are the total number of the positive cases. Immunostaining of CD70 (score 4), CD5 and CD117 in TSCC **(E)**. In contrast, thymoma showed the absence of CD70 expression **(F)**. The CD70 score in lung squamous cell carcinoma was relatively low.

The immunoactivity of CD70 was detected as coarse-granular staining in scattered medullary epithelial cells or dendritic cells, which are indistinguishable from each other morphologically, of the adult thymus (**Figures 1A, B**). In contrast, thymocyte and epithelial cells in the cortex or subcortex did not show CD70 staining.

#### IHC Analysis in TSCC, Thymoma, and LSCC

The immunostaining of CD70 in TSCC is shown in **Figure 1C**. Most TSCC (87%, 27/31) showed a score ≥1 for positivity: score 1: 12 cases (39%); score 2: four cases (13%); score 3: eight cases (26%); and score 4: three cases (10%) (**Table 2** and **Figure 1C**). The majority of CD70-positive TSCC revealed a coarse-granular pattern similar to that observed in the normal thymus. A minority of TSCC showed both granular and membranous staining patterns. CD5- and CD117-positive cases accounted for 87% (27/31 patients) and 90% (28/31 patients) of TSCC, respectively (**Figures 1D, E**). Twenty-nine cases (94%) were positive for at least two of the markers.

**Table 2:** Immunohistochemical analysis of CD70 in surgical specimens.

	TSCC	Thymoma	Thymic carcinoid	LSCC
	(n = 31)	(n = 60)	(n = 3)	(n = 30)
**CD70-positivity**	27 (87%)	0/60 (0%)	0/3 (0%)	6/30 (20%)*
Score 0	4 (13%)	60 (100%)	3 (100%)	24 (80%)
Score 1	12 (39%)	0 (0%)	0 (0%)	4 (13%)
Score 2	4 (13%)	0 (0%)	0 (0%)	2 (7%)
Score 3	8 (26%)	0 (0%)	0 (0%)	0 (0%)
Score 4	3 (10%)	0 (0%)	0 (0%)	0 (0%)

TSCC, thymic squamous cell carcinoma; LSCC, lung squamous cell carcinoma.

*The proportion of CD70-positive cases in LSCC was significantly lower than that observed in TSCC (p<0.0001).

On the other hand, all cases of thymoma and thymic carcinoid were negative for CD70. Six of 30 LSCC were CD70-positive; however, the score was ≤2 and the staining pattern showed intratumoral heterogeneity (**Figure 1F**).

In addition, the diagnostic utility of CD70 IHC analysis in small biopsy specimens were assessed, because score 1 positivity was observed in as many as 39% of operative specimens. A large proportion of TSCC was immunoreactive for CD70 (81%), CD5 (91%), and CD117 (100%). Six patients underwent both biopsy and resection of TSCC, and all specimens (biopsy and resection) were CD70-positive (**Table 3**). On the other hand, only one biopsy specimen of LSCC was positive for CD70 (8.3%), and the positivity was significantly lower compared to TSCC (p < 0.001).

**Table 3:** CD70, CD5, and CD117 immunostaining concordance between surgical and biopsy specimens.

	CD70 (%)	CD5 (%)	CD117 (%)
Positivity in biopsy specimens of TSCC	9/11 (81)	10/11 (91)	11/11 (100)
Concordance between surgical and biopsy specimens from the same patient	6/6 (100)	6/6 (100)	6/6 (100)

TSCC, thymic squamous cell carcinoma.

### Gene Expression Analysis of CD70 by RT-PCR

**Figure 2:**
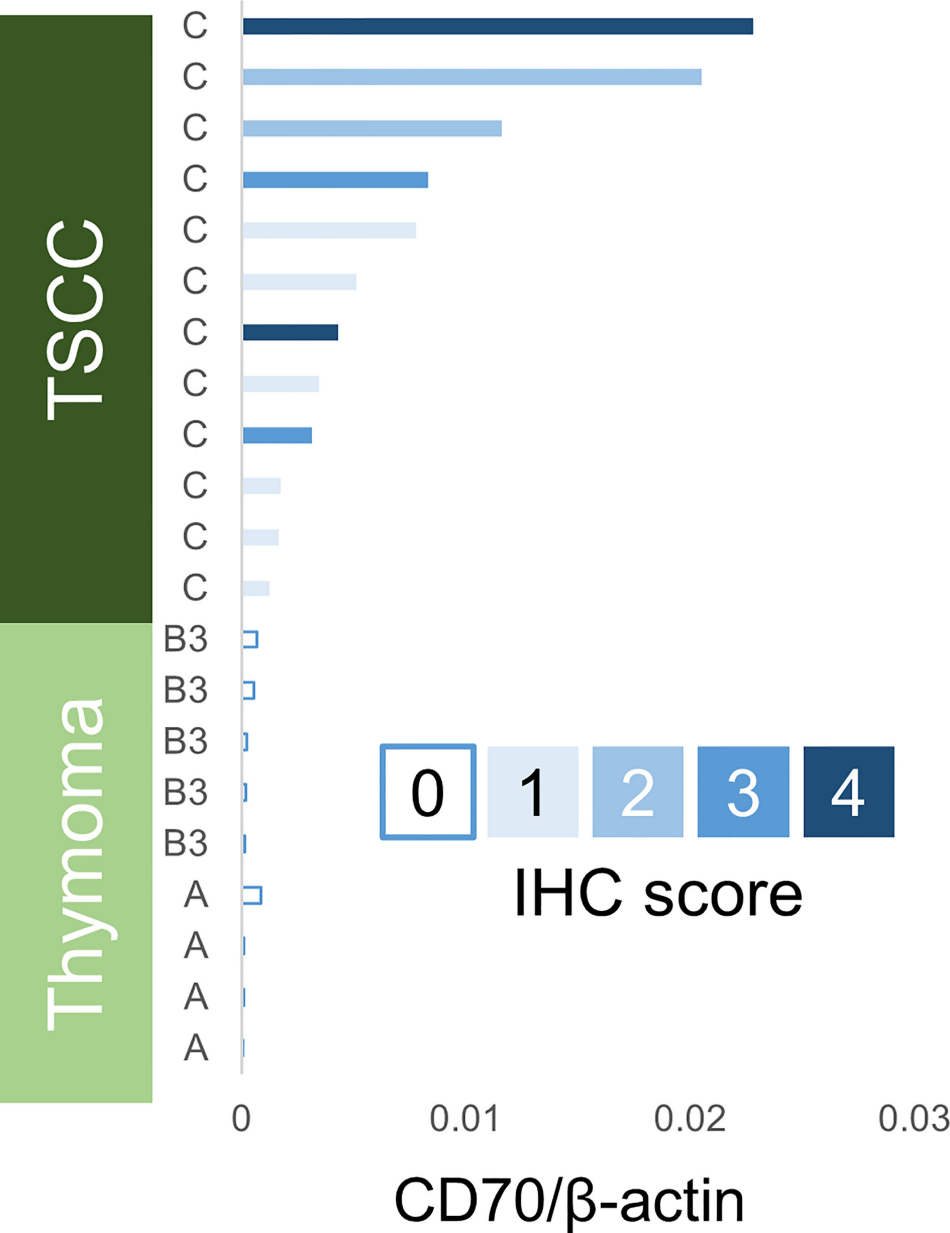
CD70 expression analysis using real-time reverse transcriptase polymerase chain reaction (RT-PCR). A, type A thymoma; B3, type B3 thymoma; C, thymic squamous cell carcinoma; TSCC, thymic squamous cell carcinoma.

The mRNA extracted from frozen tissues of TSCC (n = 12) and thymoma (type A: n = 4; type B3: n = 5) was quantified using RT-PCR for CD70 (**Figure 2**). The expression levels of CD70 in TSCC were significantly higher than those observed in thymoma (p < 0.001). This finding was consistent with the IHC score.

### Association Between Tumor CD70/PD-L1 Expression and FOXP3/CD27-Positive Lymphocytes

PD-L1 was positive (≥1%) in 74% of TSCC (**Supplementary Table 1**). Tumors with high PD-L1 score tended to have high CD70 score (p = 0.013). Some tumors were diffusely positive for both PD-L1 and CD70; however, distribution of PD-L1 positive cells and that of CD70 positive cells did not seem correlated.

**Figure 3:**
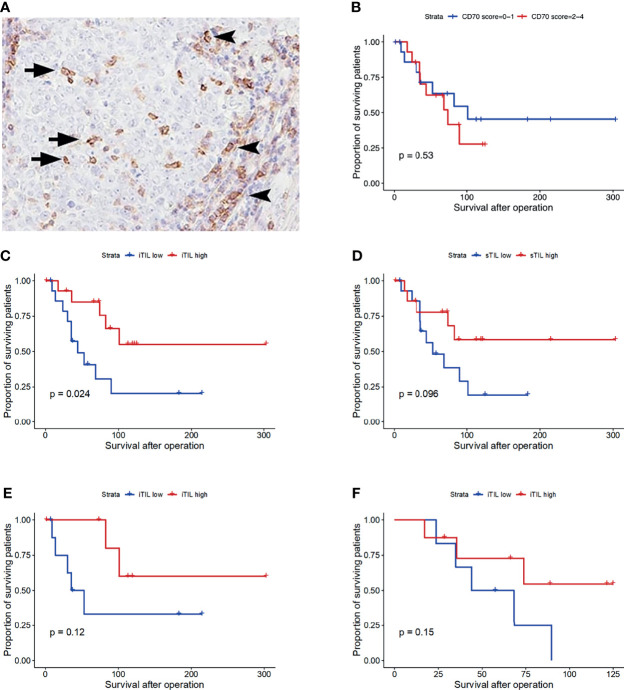
CD27 staining of thymic squamous cell carcinoma **(A)**. CD27-positive intratumoral tumor-infiltrating lymphocytes (iTIL, arrow) and stromal TIL (sTIL, arrowhead). Overall survival of patients with thymic carcinoma according to the CD70 expression status **(B)**, the number of CD27-positive iTIL **(C)** and sTIL **(D)**. Stratified survival curve in the CD70-low group **(E)** and CD70-high group **(F)**. x-axis: days.

CD27 was scantly stained in a small proportion of lymphocytes infiltrating the thymoma. In contrast, in TSCC, CD27-positive lymphocytes were observed in tumor nests and rather prominent in the fibrous stroma (**Figure 3A**). Correlation between the number of CD8-positive TIL and that of CD27-positive TIL, and correlation between the number of FOXP3-positive TIL and that of CD27-positive TIL were observed (**Supplementary Table 2**). Neither the number of CD8, FOXP3, nor CD27-positive TIL showed difference between CD70-high and low tumor, whereas the number of FOXP3-positive iTIL in CD70-high TSCC was slightly higher than CD70-low (p = 0.07, **Supplementary Table 3**). On the other hand, significant correlation was observed between PD-L1 score and the level of CD27-positive iTIL (**Table 4**).

**Table 4:** Correlation between CD27-positive TIL and PD-L1 expression.

	CD27+ iTIL			CD27+ sTIL		
	low	high	p-value	low	high	p-value
**PD-L1**			0.033			0.719
Score 0	7	1		4	4	
Score 1	7	9		9	7	
Score 2	1	5		2	4	

iTIL, intratumoral tumor-infiltrating lymphocytes; sTIL, stromal tumor-infiltrating lymphocytes; PD-L1, programmed death ligand-1.

### Survival Analysis of Patients With TSCC

We compared the postoperative survival of patients with TSCC between the CD70-high and CD70-low groups. Patient characteristics are shown in **Supplementary Table 4**. The median survival time in the CD70-high and CD70-low groups was 74 and 101 months, respectively. There was no significant difference in survival observed between the two groups (p = 0.53, **Figure 3B**).

On the other hand, patients with CD27-positive iTIL-high tumors were associated with longer survival than those with iTIL-low tumors (p = 0.024, **Figure 3C**). Moreover, the CD27+ sTIL-high and -low groups showed a slight, non-statistically significant difference in the survival curve (p = 0.096, **Figure 3D**).

PD-L1 expressional score neither showed potential for stratification of survival in the cohort (median overall survival: 74.0 months in score 0, 68.5 months in score 1, and 89.7 months in score 2, p=0.97).

The univariate Cox proportional hazard model for all clinicopathological variables revealed that the Masaoka–Koga stage IV, FOXP3-positive sTIL and CD27-positive iTIL status showed p-value <0.10 (p = 0.022, p = 0.008 and p = 0.033, respectively) (**Supplementary Table 5**), while high PD-L1 expression (≥50%) did not show significant correlation with survival (p = 0.88). Neither Masaoka–Koga stage IV, FOXP3-positive sTIL or CD27-positive iTIL status was shown to be statistically significant in the multivariate analysis (p = 0.24, p = 0.36 and p = 0.73, respectively) (**Supplementary Table 6**).

Preferable survival of iTIL-high tumor seemed more obvious in the CD70-low group than in the CD70-high group. However, the difference observed between these two subsets was not statistically significant (p = 0.12 and p = 0.15, respectively) (**Figures 3E, F**).

## Discussion

In this study, using IHC analysis of FFPE tissues, we showed that CD70 was expressed in 87% of TSCC. The percentage of TSCC with >50% (score 3 and 4, 36%) was consistent with a recent study by Flieswasser et al., whereas the sample size of our study is six times as large (34). In contrast, all cases of thymoma were negative for CD70. We also demonstrated good agreement with IHC score and mRNA expression levels of CD70 by quantitative RT-PCR in TSCC and thymoma. These results indicate that CD70 can be utilized as a specific marker discriminating TSCC from thymoma.

The IHC analysis revealed the characteristic staining pattern (coarse-granular or membranous) observed in CD70 (**Figure 1**). Notably, CD70 has been shown to be expressed in the thymic medulla (3, 20). The current study showed a consistent expression of CD70 in the normal thymic medulla adjacent to the tumor, which was also retained in <1% of tumor cells observed in the medullary island of type B1 thymoma. Thus, CD70 is one of the specific markers of medullary differentiation, along with CD40 and claudin-4 (35).

### Diagnostic Potential of CD70 IHC

Positivity for CD5 and CD117 was similar to that observed for CD70. The present findings are consistent with those reported in our previous study involving IHC analysis of CD70 in frozen sections (20). All cases of thymoma were negative for CD70. Previous studies showed that 0–7% and 0–18% of thymoma were positive for CD5 and CD117, respectively (9, 10, 12, 13, 16, 18, 36). Although the percentage of CD70 positive cells in each case was rather small, the sensitivity and specificity of CD70 in distinguishing TSCC from thymoma are comparable with those of CD5 and CD117.

In the present study, 20% of LSCC cases were positive for CD70. This percentage is similar to that reported in a previous study of LSCC (27%) (6). CD5 and CD117 are utilized as diagnostic markers of TSCC; however, LSCC occasionally expresses CD5 (0–15%) or CD117 (6.2–20%) (18, 37). In the present study, we observed one CD70-positive and CD5/CD117-negative case. These findings indicate that each marker does not independently enable complete discrimination between TSCC and LSCC. Combining IHC analyses of CD70, CD5, and CD117 might enhance the diagnostic ability, while it needs larger scale studies to confirm.

Morphological analysis using biopsy specimens is sometimes limited due to the amount of tissue. In this study, all six biopsy specimens from the patients whose surgical specimens was CD70-positive were also positive for CD70. In addition, the CD70-positivity in biopsy specimens of LSCC was significantly lower than that of TSCC. Although the sample size was small, these results may imply that adding CD70 to the IHC panel has a potential to improve the specificity and sensitivity of TSCC diagnosis, especially when biopsy is the only source of tumor tissue.

### Biological Meaning of CD70 and CD27 Expression in TSCC Tissue

The CD27-positive iTIL-high group exhibited significantly longer overall survival than the CD27-positive iTIL-low group. The sTIL-high and -low groups also showed a similar difference. These results are consistent with those of previous studies showing a favorable prognosis in solid tumors, including thymic carcinoma, with high numbers of TIL (24–30, 38). The number of CD8-positive TIL, on the other hand, was not significant prognostic factor in the univariate analysis. The effector T-cell response associated with CD27-positive TIL might partially explain the phenomenon as previously discussed (31). We also showed that the survival curves of the CD27-positive iTIL-high and -low groups became closer in the CD70-high subset. This trend suggests that CD70 expressed in tumor tissue represses anti-tumor immunity *via* CD27. In the meantime, CD27 is known to be cleaved off when interacted by CD70 (32). Therefore, another possible explanation for the trend and for the irrelevance between CD70 score and CD27+TIL is that CD27-positive iTIL-low subset of CD70-high cases may be a mixture of those with TIL-low cases and with activated CD27-CD70 axis.

Previous studies revealed the aberrant expression of CD70 in several hematological malignancies and solid malignant tumors, including lung cancer, renal cell carcinoma, glioblastoma osteosarcoma, nasopharyngeal carcinoma and ovarian carcinoma (6, 39–44),. The role of the CD70–CD27 axis in anti-tumor immunity is controversial. In physiological conditions, CD70 is expressed transiently in lymphocytes and dendritic cells and affects its receptor, CD27, leading to differentiation into effector or memory T-cells (2). On the other hand, the constitutive expression of CD70 observed in chronic viral infection and lymphoma leads to immune exhaustion (45, 46). Moreover, the expression of CD70 in renal carcinoma cell lines induces apoptosis of lymphocytes (47). We could not detect the significant difference between CD70-high and -low tumor in the levels of CD8-positive TIL or FOXP3-positive TIL; however, the number of FOXP3-positive iTIL in CD70-high TSCC was marginally larger than CD70-low TSCC. This result may reflect activation of CD70–CD27 axis and subsequent maintenance of regulatory T-cells. The impact of the amount of such TILs in TSCC to patient survival is still unclear. We suspect that CD70-expressing tumor induces immune suppressed state *via* CD70–CD27 pathway, which warrants further studies to confirm.

### CD70 as Immunotherapeutic Target

Some anti-CD70 reagents have been developed for cancer. For example, SGN-CD70A underwent phase I trial in CD70-positive renal cell carcinoma patients (48). In addition, CD70 expression on malignant pleural mesothelioma and lower CD27–positive TIL accumulation are reported to correlate with poor prognosis (49). The present study showed that the survival curves of the CD27-positive iTIL-high and -low groups became closer in the CD70-high subset. This implies that CD70-targeted reagent may be beneficial for TSCC patients.

PD-L1 expression levels in thymic epithelial tumors and the correlation between survival have been examined in some studies but the results varied (50–53). Our study demonstrated no evident correlation between PD-L1 score and survival. It may be derived from small sample size, while PD-L1 expression alone might not be prognostic factor in thymic carcinoma patients who did not receive immunotherapy.

Patients with thymoma/thymic carcinoma have been shown that those with high-PD-L1 expression (>50% of tumor cells) appeared to have responded better to pembrolizumab (an anti-PD-L1 reagent), compared to those with low-PD-L1 expression (21, 22). Our result showed that CD70 and PD-L1 expression is correlated in TSCC. Therefore, combination of anti-CD70 and anti-PD-L1 reagents may be beneficial for many of the TSCC patients with high-PD-L1 expression. In addition, low-PD-L1 expression TSCC tended to have lower CD70 score and lower number of CD27-positive iTIL. In such cases, CD27-agonsitic treatment which is expected to facilitate immunity may be beneficial.

Chimeric antigen receptor (CAR) T cell therapy has achieved great success in the　treatment of several hematologic malignancies. Recent evidence revealed that CAR T cells targeting CD70 could inhibit the growth of glioma (54), head and neck squamous cell carcinoma (55), and multiple solid tumors (56) as well as hematologic malignancies (57) *in vitro* and *in vivo*. CD70-targeting CAR T cell therapy may also be a new candidate for immunotherapy in thymic carcinoma.

### Limitation*s*

There were some limitations to our study. First, TSCC is a rare type of cancer; thus, the number of analyzed patients was small. Additional replication and collaboration studies are required. The multivariate analysis failed to show an independent survival effect of TIL or Masaoka–Koga staging, which also might partially be due to the small number of patients. Studies involving larger numbers of cases are warranted to assess the prognostic value of TIL. Second, sampling bias could not be excluded because we performed only IHC analysis in the representative specimens of each tumor. We confirmed the concordance between the staining pattern of CD70 in TSCC and expression using quantitative RT-PCR. Therefore, we suppose that a small amount of TSCC tissue may be sufficient to evaluate the expression of CD70.

## Conclusion

CD70 was specifically positive in TSCC versus thymoma. In addition, in TSCC, a significantly higher positivity was observed than in LSCC. The CD70–CD27 axis is a potentially useful diagnostic marker and a promising therapeutic target.

## Data Availability Statement

The raw data supporting the conclusions of this article will be made available by the authors, without undue reservation.

## Ethics Statement

This study was approved by the Institutional Review Board of the Tokyo Metropolitan Cancer and Infectious Diseases Center Komagome Hospital (approval number: 2120). Written informed consent for participation was not required for this study in accordance with the national legislation and the institutional requirements.

## Author Contributions

JK and TH drafted the manuscript. JK, TH, MO, YH, SH, TM, TU, and MF contributed to histopathological and immunohistochemical analysis. JK, YO, and HH contributed to the analysis of clinicopathological features. All authors contributed to the article and approved the submitted version.

## Conflict of Interest

The authors declare that the research was conducted in the absence of any commercial or financial relationships that could be construed as a potential conflict of interest.

## Publisher’s Note

All claims expressed in this article are solely those of the authors and do not necessarily represent those of their affiliated organizations, or those of the publisher, the editors and the reviewers. Any product that may be evaluated in this article, or claim that may be made by its manufacturer, is not guaranteed or endorsed by the publisher.

## Abbreviations

FFPE, formalin-fixed, paraffin-embedded; RT-PCR, reverse transcriptase polymerase chain reaction; IHC, immunohistochemical; ITMIG, International Thymic Malignancy Interest Group; TIL, tumor-infiltrating lymphocytes; iTIL, intratumoral tumor-infiltrating lymphocytes; sTIL, stromal tumor-infiltrating lymphocytes, PD-L1, Programmed death ligand-1; HE, hematoxylin and eosin.
