# Supplementary material for: CD70 in Thymic Squamous Cell Carcinoma: Potential Diagnostic Markers and Immunotherapeutic Targets
Source: Front Oncol. 2022 Jan 25;11:808396. doi: 10.3389/fonc.2021.808396 (PMC8821901; doi:10.3389/fonc.2021.808396)
Supplement: Supplementary file 1 [file Table_1.docx]

Supplementary Table 1. Relation of positivity score between CD70 and PD-L1 in TSCC

|  |  | PD-L1 |  |  | p |
| --- | --- | --- | --- | --- | --- |
|  |  | Score 0 | Score 1 | Score 2 | 0.013 |
| CD70 | Score 0 | 3 | 0 | 1 |  |
|  | Score 1 | 4 | 7 | 1 |  |
|  | Score 2 | 1 | 1 | 2 |  |
|  | Score 3 | 0 | 7 | 1 |  |
|  | Score 4 | 0 | 1 | 2 |  |

PD-L1: programmed death ligand-1; TSCC: thymic squamous cell carcinoma

Supplementary Table 2. Correlation between CD8, FoxP3 and CD27-positive TIL

| Combination |  | Rho | p |
| --- | --- | --- | --- |
| Intratumoral TIL |  |  |  |
| CD8+ iTIL | FOXP3+ iTIL | 0.80 | <0.0001 |
| FOXP3+ iTIL | CD27+ iTIL | 0.63 | 0.00017 |
| CD27+ iTIL | CD8+ iTIL | 0.65 | 0.00010 |
| Stromal TIL |  |  |  |
| CD8+ sTIL | FOXP3+ sTIL | 0.74 | <0.0001 |
| FOXP3+ sTIL | CD27+ sTIL | 0.62 | 0.00025 |
| CD27+ sTIL | CD8+ sTIL | 0.60 | 0.00047 |

Supplementary Table 3. The number of TIL in CD70-low and -high TSCC (/mm^2^) (median, [range])

|  | CD70-low | CD70-high | p-value |
| --- | --- | --- | --- |
| CD8-positive iTIL | 308 [6-1921] | 472 [83-2475] | 0.37 |
| CD8-positive sTIL | 1132 [233-3564] | 1810 [466-3003] | 0.22 |
| FOXP3-positive iTIL | 100 [6-750] | 212 [28-883] | 0.07 |
| FOXP3-positive sTIL | 392 [44-2203] | 753 [58-2555] | 0.20 |
| CD27-positive iTIL | 92 [1-1578] | 179 [5-629] | 0.69 |
| CD27-positive sTIL | 1184 [87-5586] | 1117 [126-3553] | 0.84 |

Supplementary Table 4. Characteristics of patients with TSCC

|  | CD70-low | CD70-high | p-value |
| --- | --- | --- | --- |
| **Male/female** | 10/6 | 7/7 | 0.713 |
| **Age, years (median, range)** | 68, (41–83) | 61.5, (44–77) | 0.240 |
| **Masaoka–Koga staging** |  |  | 0.414 |
| I | 0 | 1 |  |
| II | 3 | 4 |  |
| III | 7 | 2 |  |
| IVa | 1 | 2 |  |
| IVb | 5 | 5 |  |
| **CD27+ iTIL** |  |  | 0.715 |
| Low | 9 | 6 |  |
| High | 7 | 8 |  |
| **CD27+ sTIL** |  |  | 0.715 |
| Low | 7 | 8 |  |
| High | 9 | 6 |  |
| **PD-L1** |  |  | 0.013 |
| Score 0 | 7 | 1 |  |
| Score 1 | 7 | 9 |  |
| Score 2 | 2 | 4 |  |

TSCC: thymic squamous cell carcinoma; iTIL: intratumoral tumor-infiltrating lymphocytes; sTIL: stromal tumor-infiltrating lymphocytes; PD-L1: programmed death ligand-1

Supplementary Table 5. Univariate survival analysis in patients with TSCC

|  | Hazard ratio (95% CI) | p-value |
| --- | --- | --- |
| **Male/female** | 0.96 (0.38–2.89) | 0.936 |
| **Age** | 1.00 (0.96–1.04) | 0.924 |
| **Masaoka–Koga stage IV** | 3.43 (1.20–9.83) | 0.022 |
| **CD70-high** | 1.39 (0.50–3.89) | 0.528 |
| **CD8+ iTIL-high** | 0.64 (0.23–1.83) | 0.41 |
| **CD8+ sTIL-high** | 0.62 (0.22–1.72) | 0.36 |
| **FOXP3+ iTIL-high** | 0.48 (0.17–1.36) | 0.17 |
| **FOXP3+ sTIL-high** | 0.22 (0.07–0.67) | 0.008 |
| **CD27+ iTIL-high** | 0.30 (0.10–0.91) | 0.033 |
| **CD27+ sTIL-high** | 0.41 (0.14–1.12) | 0.107 |
| **PD-L1 high** | 0.95 (0.26–2.24) | 0.884 |

TSCC: thymic squamous cell carcinoma; CI: confidence interval; iTIL: intratumoral tumor-infiltrating lymphocytes; sTIL: stromal tumor-infiltrating lymphocytes

Supplementary Table 6. Multivariate survival analysis in patients with TSCC

|  | Hazard ratio (95% CI) | p-value |
| --- | --- | --- |
| **Masaoka–Koga stage IV** | 2.07 (0.62–6.97) | 0.24 |
| **FOXP3+ sTIL-high** | 0.39 (0.06–2.83) | 0.36 |
| **CD27+ iTIL-high** | 0.73 (0.13–4.25) | 0.73 |

iTIL: intratumoral tumor-infiltrating lymphocytes
